# Building Capacity of Public Health Workforce in the Global South Through Humphrey Fellowship

**DOI:** 10.3389/phrs.2025.1608111

**Published:** 2025-01-24

**Authors:** Monzur Morshed Patwary, Sabanur Çavdar, Heba Metwally, Eman Salih, Richard Kambarangwe

**Affiliations:** ^1^ CWW, Dhaka, Bangladesh; ^2^ Eyupsultan District Health Directorate, Turkish Ministry of Health, Istanbul, Türkiye; ^3^ Department of Public Health, School of Medicine, Istanbul Medipol University, Istanbul, Türkiye; ^4^ Egypt Healthcare Authority, Cairo, Egypt; ^5^ School of Public Health, Yale University, New Haven, CT, United States; ^6^ Tanzania Health Promotion Support (THPS), Masaki, Tanzania

**Keywords:** global south, public health, capacity building, fellowship, mid-career, professional development, hubert humphrey, global health

## Introduction

Practitioners from the global south have demonstrated incredible resilience and adaptability in addressing public health crises like Ebola and COVID-19 [1]. Despite lacking substantial resources, they have implemented localized solutions which garnered universal acclaim. Yet, they face significant challenges due to limited access to education and professional development opportunities compared to others. This disparity is often worsened by inequalities that curb the capacity for capacity development [2]. Investing in the skills of public health workers and building partnerships improve the capacity of local health systems to respond to emergencies. Bolstering these capacities through fellowships and collaborative initiatives can bridge the gap between the Global North and South, leading to more equitable health outcomes worldwide.

There already exists a number of fellowship programs in the area of global health. For example, the HGHI Burke Climate and Health Fellowship program supports professionals with research at the intersection of climate change and global health [3]. The Global Surgery Fellowships aims to improve access to surgical care worldwide [4]. The Public Health Informatics Fellowship Program (PHIFP) offered by CDC focuses on building a robust team of health informatics professionals across the globe [5]. Atlantic Fellows for Health Equity is another US-based fellowship that is focused around for professionals interested in the field of health equity [6]. The NIHR Global Advanced Fellowship supports postdoctoral researchers in advancing their careers in global health research [7].

The Humphrey Fellowship Program discerns itself from other fellowships by offering a non-degree, tailored experience for mid-career professionals. Unlike research-focused programs, it combines academic coursework with flexible professional development opportunities sans the rigors of pursuing a traditional degree. Post-fellowship, individuals return to their native country with a global network and improved skills to come up with effective solutions to health challenges. This article discusses the program’s unique structure, the transformative journeys of fellows, and their contributions to global health.

## Structure of the Hubert H. Humphrey Fellowship

The Hubert H. Humphrey Fellowship Program was initiated in 1979 by the former United States President Jimmy Carter to honor the late senator and former US vice president Hubert H. Humphrey. The program brings mid-career professionals from 163 countries (mostly from the global south) to the United States for a duration of 10 months [8]. The program is administered by the Institute of International Education and funded by the U.S. Department of State. Among the impact areas of the program, “Public Health Policy and Management” is one that has really stood out over the years.

The individuals selected for this fellowship at partner institutions such as Emory University, Virginia Commonwealth University (VCU), Tulane University, and Johns Hopkins University (JHU) have not necessarily followed a linear path in public health, but often came from a diverse path.

## Tailored Learning Through Classroom Experiences and Skill-Building Workshops

At host universities, fellows have the opportunity to take graduate-level courses on pandemic preparedness, one health, non-communicable diseases, nutrition, transforming surveillance and many other topics. However, as Elisaveta Petrova-Geretto (Emory University, 2021–2022) attested, fellows may also take classes from other departments if there is an interest.

Additionally, Humphrey Seminars provides fellows with weekly sessions on timely public health issues led by subject matter experts. It also strengthens the leadership and technical capacity through immersive training workshops typically held in different US cities. Eman Salih (Emory University, 2022–23) from Sudan underlined the life-saving guidance she could offer during a national crisis due to her crisis leadership training. Dr. Bani (JHU, 1988–1989) from Sudan credits the fellowship’s leadership training with preparing him for high-responsibility roles like the Deputy Head of the Nutrition Sector for all UN agencies in Darfur.

## Mentorship

Early in their fellowship, individuals are connected with a mentor who facilitates the process of finding potential collaborators within host academic institutions and beyond, during and even after the fellowship. Regarding mentorship, Dr Bani said, “I worked closely with my mentor, Professor Timothy Baker to design my program for the year. It was an incredibly enriching experience.”

## Field Visits

The program has collaborated with various organizations which range from federal agencies like CDC to small volunteer organizations. Direct interaction with C-suite executives and directors at these organizations through weekly visits offers insights far beyond the standard student experience.

## Professional Affiliation

Fellows at Emory University (presently the sole host institution) benefit from the institution’s location at the “public health capital of the world,” Atlanta which is home to US Centers for Disease Control and Prevention (CDC), departments of public health, and well-known non-profit organizations.

**FIGURE 1:**
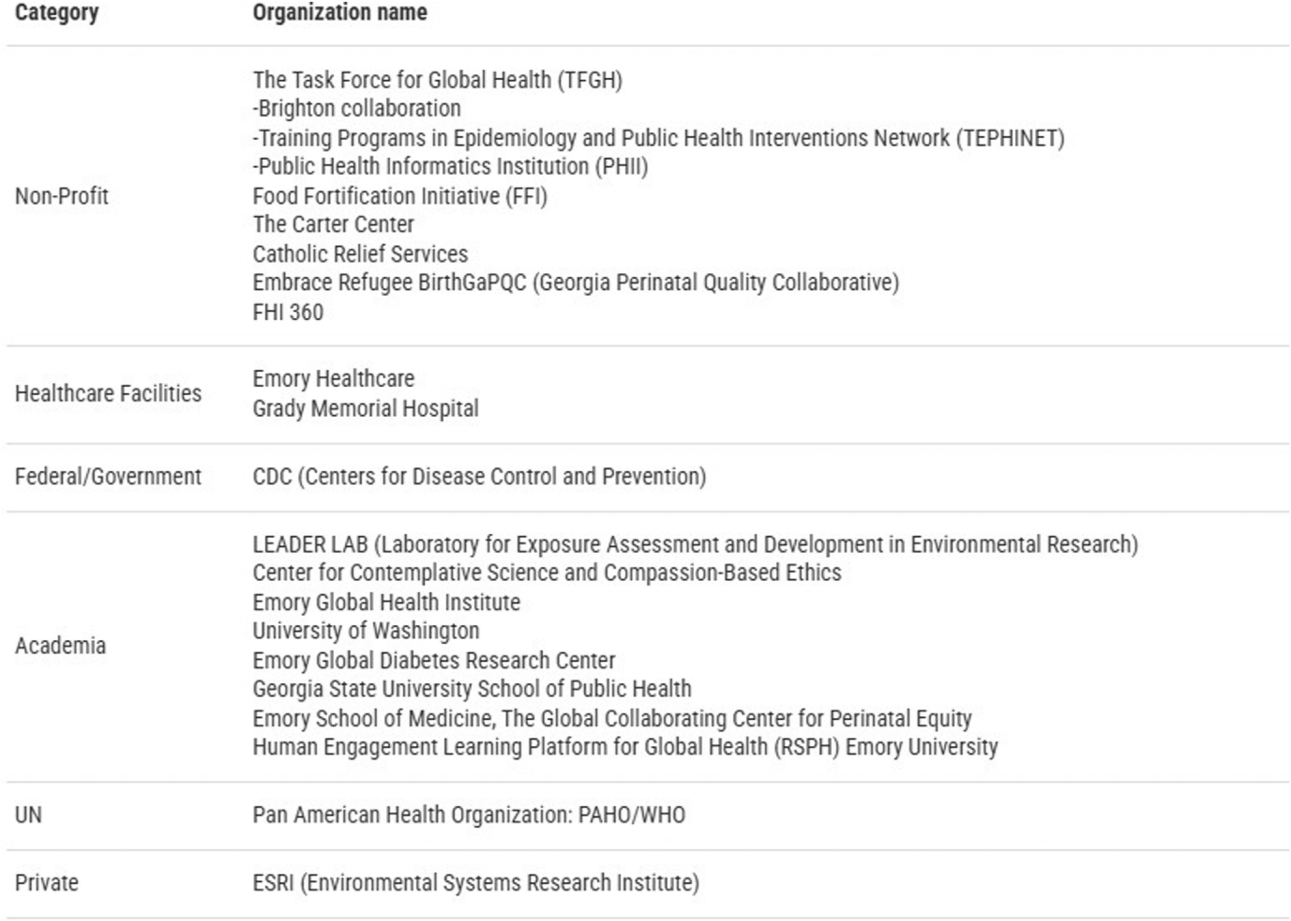
Organizations that have regularly hosted Humphrey Fellows for their professional affiliation in areas of global health *(Compiled by the authors).*

Through the professional affiliation component, fellows engage in part-time work with US-based organizations (Figure 1). Several fellows have gone on to foster long-lasting partnerships with them.

Building on her professional affiliation at the Focus Area for Compassion and Ethics (FACE) program, Elisaveta is presently researching informed consent in public health with the focus of vaccination consent. She also plans to host compassion seminars and discuss compassion in healthcare in three hospitals jointly with the FACE team who will travel to Bulgaria. She is also planning on organizing global health-themed case competitions, learning from her experience at Emory University. Furthermore, she has also begun working on an LGBTQIA+ guidebook for inclusive language in healthcare.

Through her Professional Affiliation, Daisy Vargas Méndez from Panama (VCU, 2022–2023) learned about motivational interviewing, a crucial skill for professionals in mental health and counseling. Another big takeaway for her was the understanding of the operations and management at recovery centers in the United States.

Henry Espinoza (VCU, 2022–23) worked with a local VCU-based lab conducting research on mental health and substance use. He explored and validated different tools and reviewed literature to understand the project. Now working with a local NGO in Nicaragua, he applies the skills gained during his time in Virginia. His career, primarily focused on sexual and reproductive health, has now expanded to include substance use during pregnancy. This comprehensive expertise makes him highly competent in addressing these critical issues.

Dr. Ibrahim Bani’s professional affiliation experience at the World Bank helped him develop networking skills and leverage many opportunities. He says, “After the fellowship, I collaborated on a micronutrient deficiency project in Darfur, which was supported by Nutrition International. We developed a premix product containing essential nutrients for women and children in displaced communities. The project was a success and was presented at various international conferences.”

## Continued Capacity Building Opportunities and Fellowship-Supported Project

Even after the fellowship duration, fellows are supported in their endeavors to upskill and implement impactful projects in their field. The Professional Development Grant enables fellows to attend conferences/workshops to further develop their skills and networks while the Alumni Impact Award (AIA) encourages fellows to pursue relevant projects in their home country. Richard, through the latter, implemented a project aimed at promoting mental health awareness among university students in Kilimanjaro, Tanzania while also facilitating youth-focused activities in the intersection of climate change and health.

## Conclusion

The platform empowers fellows to make significant contributions to the enhancement of global health outcomes. By building the capacity of the global health workforce around the world, the Humphrey Fellowship program catalyzes positive change and fosters lasting impact in communities worldwide.
